# Editorial: Rare forms of pediatric adrenal disorders: beyond congenital adrenal hyperplasia due to 21-hydroxylase deficiency

**DOI:** 10.3389/fendo.2024.1512883

**Published:** 2024-12-11

**Authors:** Rosario Ferrigno, Mariacarolina Salerno, Martin O. Savage

**Affiliations:** ^1^ UOSD di Auxologia ed Endocrinologia, AORN Santobono-Pausilipon, Naples, Italy; ^2^ Pediatric Endocrine Unit, Department of Translational Medical Sciences, University of Naples Federico II, Naples, Italy; ^3^ Centre for Endocrinology, William Harvey Research Institute, Barts & the London School of Medicine & Dentistry, Queen Mary, University of London, London, United Kingdom

**Keywords:** adrenal diseases, childhood, adrenal insufficiency, Cushing’s syndrome, pheochromocytoma, adrenocortical carcinoma

Defects of the adrenal gland in childhood comprise a broad spectrum of etiologies, ranging from genetic variants resulting in adrenal insufficiency (1), autoimmune dysfunction (1), impaired stimulation by the hypothalamic-pituitary axis (1), and adrenal tumors (2–4) that may result in autonomous neoplastic hypersecretion of both adrenal cortical (3) and medullary steroids (4). Adrenocortical hypofunction can be a life-threatening condition requiring urgent diagnosis and replacement with glucocorticoids (1). In contrast, hypersecretion of cortisol as in Cushing’s syndrome leads to specific clinical features such as growth failure, obesity, hirsutism and osteoporosis, which if undiagnosed can cause major morbidity and decrease in quality of life (3). Adrenal tumors, including adrenocortical carcinomas and pheochromocytomas, may be isolated or a component of familial endocrine neoplastic syndromes, requiring urgent diagnosis, genetic characterization, and surgical removal (2–4).

In this collection of articles, we describe the rare genetic causes of adrenal insufficiency and discuss the diagnosis, molecular characterization, and treatment of adrenal defects that comprise the diverse landscape of familial glucocorticoid deficiency syndromes (Liu et al., Maharaj). Autoimmune Addison’s disease is also discussed which, although rare in childhood, may occur in isolation or as a component of polyendocrine autoimmune dysfunction in conjunction with hypoparathyroidism and gonadal insufficiency (Capalbo et al.). The molecular features are also described (Capalbo et al.). The genetic origins of adrenoleukodystrophy are discussed together with the pediatric features, therapeutic approaches and long-term prognostic predictions (Cappa et al.).

Finally, neoplastic disorders such as adrenocortical carcinoma, Cushing’s syndrome, and pheochromocytoma are discussed in detail (Unsal et al., Zagojska et al., Guarnotta et al., Savage et al., Bima et al.). Adrenocortical carcinoma is extremely rare in childhood but is associated with a poor prognosis (Zagojska et al.). Cushing’s syndrome may be ACTH-dependent, as in Cushing’s disease, which is the secretion of excess ACTH by a pituitary adenoma or ectopic ACTH syndrome, or ACTH-independent, as in adrenocortical tumor or adrenocortical hyperplasia, which may be isolated or part of the genetic complex of McCune-Albright syndrome or Carney Complex (Unsal et al., Guarnotta et al., Savage et al.). Guidelines for the management of pediatric pheochromocytoma are described together with therapeutic recommendations for the management of hypertension (Bima et al.).

Our aim in bringing together this collection of articles is to provide clinical and scientific updates to inform and educate both pediatric and adult endocrinologists and their nursing support staff about the wide range of adrenal disorders that, although rare in childhood, can be life-threatening and, by definition, serious. Collaboration between pediatric and adult endocrinology staff is emphasized, particularly in disorders such as familial endocrine neoplasia and Cushing’s syndrome, where few pediatricians have extensive experience in the management of these disorders.
